# Study on bio-inspired feet based on the cushioning and shock absorption characteristics of the ostrich foot

**DOI:** 10.1371/journal.pone.0236324

**Published:** 2020-07-24

**Authors:** Dianlei Han, Rui Zhang, Guolong Yu, Lei Jiang, Dong Li, Jianqiao Li

**Affiliations:** 1 Key Laboratory of Bionic Engineering, Ministry of Education, Jilin University, Changchun, PR China; 2 China North Vehicle Research Institute, Beijing, PR China; 3 Beijing Institute of Spacecraft Environment Engineering, Beijing, PR China; University of Memphis, UNITED STATES

## Abstract

As the main actuator of high-speed running, the ostrich feet are highly capable of cushioning and shock absorption. In this study, based on the elastic modulus scales and assembly order of the 3rd toe soft tissues and the functions of the metatarsophalangeal (MTP) joint, we designed fourteen bio-inspired feet. The impact process on loose sand was simulated on the finite element software Abaqus. Also the stress distributions and deformations of each component of the bio-inspired feet were clarified. With the peak acceleration as the index, the cushioning performances of the bio-inspired feet were compared on both loose sand and solid ground through height-variable impact tests. The 15-15-15 HA (hardness unit) bio-inspired foot showed lower peak acceleration and thereby better cushioning performance, but larger deformation, less-uniform stress distribution and thereby lower stability than the 15-35-55 HA bio-inspired foot. In fact, the silicon rubbers with different hardness degrees (which simulate the elasticity modulus scales of the digital cushions, fascia and skin) and the spring mechanism (which simulates the functions of the MTP joint) work as an “integrated system” of cushioning and shock absorption.

## Introduction

When the feet of large-weight running animals contact with the ground, the hindlimbs need to bear the ground impact. To prevent the hindlimb bones and tissues from damage or destruction, these animals should reduce ground stress waves as much as possible [1]. After long-term natural selection and evolution, excellent running animals have inevitably developed efficient cushioning and shock absorption systems to avoid injury during locomotion. This phenomenon can be illustrated by many cases. Firstly, the large subcutaneous cushions in the feet of African elephants (*Loxodonta africana*) are pivotal in distributing forces during weight-bearing and in storing or absorbing mechanical forces [2–6]. Secondly, the morphology and arrangement of collagen fiber bundles in tendons [7,8] and the rough connective tissues, mucous tissues, fibrous cartilages and adipocytes of the digital cushions in horses function together to absorb mechanical vibration [9,10]. Thirdly, the multilayer cushions of German shepherd dogs mainly consist of a stratified epithelium layer, a dermis layer and a subcutaneous layer, which work jointly in the paw pad to meet the biomechanical requirements for locomotion [11,12]. Moreover, soft tissues conduct both negative work and positive work in human walking [13,14]. The deformation of soft tissues may save muscles the active dissipating energy, and their elastic rebound can save up to 14% of the total positive work per stride.

African ostriches (*Struthio camelus*), perennially living in deserts, are capable of remarkable speed, exceptional endurance and continuous locomotion. Adult ostriches can span 3.5 to 7 m every stride and continuously run at the speed of 50–60 km/hr (sprint speed over 70 km/hr) for up to 30 min without feeling fatigue, indicating ostriches are the fastest birds in terrestrial locomotion [15–18]. These cursorial birds possess extraordinary speed and endurance, especially in deserts, and thus are an ideal large-scale animal model for mechanical study of locomotion on granular substrates. Such high-speed running ability is attributed to the muscles, tendons, legs, feet and other anatomical structures as well as their coordination [19,20].

The 3rd toe of the ostrich foot is mainly composed of skin, fascia, digital cushions, flexor tendons and phalanges in the sagittal plane [21]. After skin removal from the 3rd toe, it is clear three digital cushions are arranged in parallel and wrapped by viscoelastic fascia [22]. Optical and electronic microscopes uncover two types of adipocytes in the digital cushions of toe pad, including the typical signet ring cells with large fat droplets in dimensions dwarfing the cell organelles, and the diffused oval-shaped adipocytes [23]. Fascia-encapsulated adipocytes can absorb the ground impact by compression and rebound and thus are critical in the buffering process. The hardnesses, elastic moduli, and densities of different soft tissues (e.g. skin, fascia and digital cushions) have been measured, and excellent cushion characteristic of ostrich toe pad has been analyzed using the finite element method (FEM). The toe pad can disperse the load effectively and without excessive deformation [24]. If the internal material is too soft, the maximum impact force and stress of the external structure will be significantly improved, and the stability will be deteriorated. Therefore, the foot pad should have a moderate hardness to maintain the stability of the touchdown process [24]. An ostrich foot locomotion system composite model showed the von Mises stress maximized in bones, followed by articular cartilages and plantar soft tissues [25]. Thus, soft tissues, such as skin, fascia and digital cushions, play an important role in the cushioning and shock absorption of ostrich feet.

The connective metatarsophalangeal (MTP) joint is permanently elevated above the ground [26] and is critical in energy storage and shock absorption between the touch-down and lift-off of the foot [27]. When an ostrich foot touches the ground, the position of this joint firstly descends and then automatically rises to the original posture, which is just like spring [28]. Compared with bipedal humans, ostriches run at two-fold speeds with about 50% less metabolic cost, although the two species have similar weights and heights [19,29]. Histology and scanning electron microscopy showed the collagen fiber bundles in the proximal MTP were mainly wavy-type and likely involved in energy storage and shock absorption, those in the middle MTP were straight-type and mainly acted to change the force direction, but those in the distal MTP were not consistently arranged [30]. Significant differences in locomotion patterns were found between running and slow walking in the MTP joint of ostriches, but not in the interphalangeal joint of the 3rd or 4th toe [31–33]. Moreover, the 3rd toe as the main load-bearing element whilst the 4th toe as the complementary load-sharing element really worked as an “integrated system”, which primarily ensured the lateral stability of the permanently-elevated MTP joint [26,32,33].

As a useful and continually developing research method, bionics is more and more widely used in engineering. The basic principle of bionic engineering is how to apply the superior performance of biology to solve actual engineering problems. Therefore, simplification is often employed to make complex problems convenient, easy and practical in reality. For instance, despite the complicated limbs-ground interaction, both the walking gait and the running gait can be illustrated by spring- loaded inverted pendulum models [34,35], and the gait dynamics and thereby the oscillation modes vary largely along with different combinations of kinetic energy, leg compliance and leg contact conditions [36]. Firstly, the walking gait involves an inverted-pendulum model that accounts for efficient exchange between potential energy and kinetic energy within each step. Secondly, the rapid locomotion gait (e.g. running, hopping and galloping), in which the center of mass (COM) is inverse to the walking gait, is described as a spring-mass model that accounts for the exchange of mechanical energy. Minimization of metabolic-energy costs is a primary determinant of gait selection by terrestrial animals. Ostriches tend to select the inverted pendulum gait at low speeds and the bouncing gait at high speeds to improve locomotion performance and energy economy [37,38].

Mechanical feet, in direct contact with the ground, are the key component of a legged robot and their structure and material are two important influence factors on the cushioning and shock absorption of the legged robot on unconventional ground [39]. Many robot feet are flat and contact in large area with flat terrains. Most humanoid robots have flat and rectangular feet, such as ASIMO [40] and PETMAN [41]. A legged robot to walk or run on rugged terrains is often designed with cylindrical or spherical feet, so as to more freely adapt to the terrains, such as BigDog [42], LS3 [43] and LittleDog [44]. Some robots, especially bio-inspired structures, have irregularly-shaped feet, such as the bent foot of Sandbot robot [45]. Cotton et al. developed a fast and efficient bipedal robot, named FastRunner [46], by using rods to simulate the skeletal elements of ostrich hindlimbs and a number of springs to simulate tendons [27,28], which played a role in energy storage and shock absorption.

Many of the existing and future legged robots are designed to cross neither structured nor flat and hard field terrains. Contact mechanics is essential in the manufacture of these legged robots [47]. Kang et al. [48] and van der Meer [49] studied the response of granular matter to the intrusion of rigid objects and thereby modeled many aspects of the behavior of granular matter, including plastic flow. As we all know, there are relatively few studies on foot-soil interaction owing to the complex influencing factors, especially in the case of deformable feet on deformable terrains. Based on terramechanics, Ding et al. used a spring mechanism to simulate deformable feet and thus established a foot-terrain interaction mechanical model for legged robots [39]. Aguilar and Goldman investigated a new class of granular interactions: rapid intrusions by objects that changed shape or self-deformed through passive and active means [50]. However, they did not achieve the deformable foot from the perspective of hard and soft material assemblies. A layer of rubber is often stuck under these mechanical feet (even without any soft materials) to cushion and absorb shock. In this study, inspired by the assembly order of ostrich plantar soft tissues, we aimed to study how to assemble the plantar soft materials to improve the cushioning and shock absorption effects on loose sand.

Based on the ostrich foot anatomies and biomechanics, we tried to preliminarily apply the excellent cushioning principle to design bio-inspired feet. Regarding how the material properties and structures affected the cushioning and shock absorption performances of bio-inspired feet, we hypothesized that such performances of bio-inspired feet were affected jointly by material assemblies and special structures. Therefore, we selected silicon rubbers with different hardness degrees to model the elasticity modulus scales of soft tissues in the ostrich toe pad, and used a spring mechanism to model the functions of the MTP joint. Based on the assembly order of soft tissues and the absence or presence of the spring mechanism, we designed fourteen bio-inspired feet and compared their cushioning and shock absorption performances by combining impact tests and FEM.

## Materials and methods

### Design of bio-inspired feet

**Fig 1:**
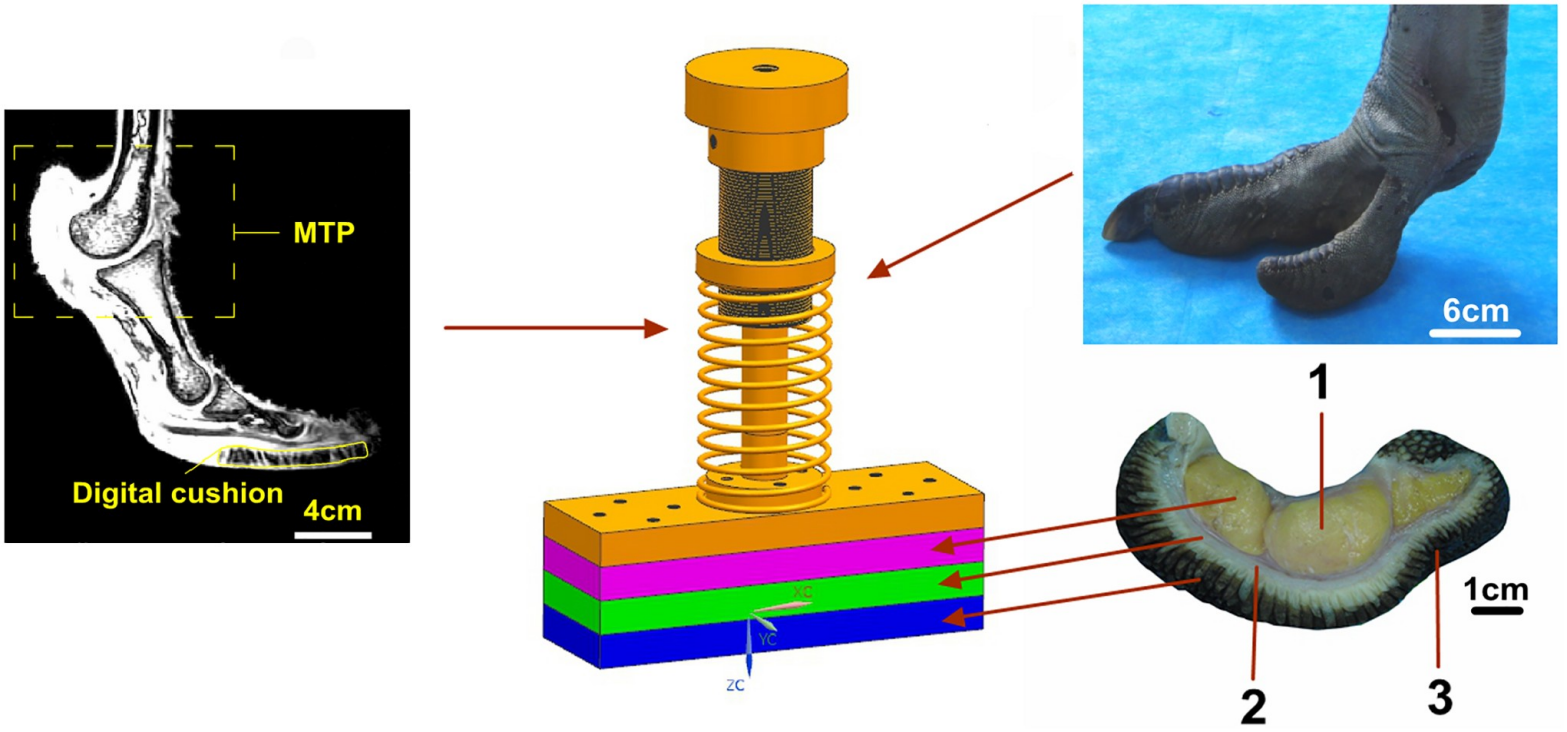
Design rationale of a bio-inspired foot. 1—digital cushions, 2—fascia, and 3—skin [53].

The 3rd toe was chosen as a bionic prototype, and the assembly order and structures of different soft tissues were simplified according to the principle of bionic engineering. Thus, the 3rd toe was mainly composed of skin, fascia, digital cushions, flexor tendons and phalanges on the sagittal plane from bottom to top (Fig 1). Flexor tendons, with a much higher elastic modulus [51,52] than the digital cushions, fascia and skin, can be regarded as hard materials. Therefore, the flexor tendon layer and the phalange layer were integrated into one layer, which was simulated with a 45# steel plate. Moreover, the foot skin layer, the fascia layer, the digital cushion layer and the phalange layer were simplified to the same thickness.

The hardness levels, elastic moduli, and densities of different soft tissues, including skin, fascia and digital cushions, were measured and published [24]. Here, test details were not repetitively described and related parameters were directly cited. In addition, the Poisson's ratios of ostrich foot digital cushions, fascia and skin were also directly cited from the other published papers [54] (Table 1).

**Table 1:** Mechanical parameters of ostrich foot tissues. Densities, elasticity moduli (E_0_) and Poisson's ratios of ostrich foot flexor tendons, digital cushions, fascia and skin. A spring mechanism was used to simulate the functions of the MTP joint, while the stiffness coefficient (K_0_) was 100 N·mm^-1^.

	Density (g·cm^-3^)	E_0_ (MPa)	Poisson's ratio	Data sources
Flexor tendons	1.12	1500.00	0.30	[51,52]
Digital cushions	7.30×10^−4^	3.80	0.47	[24,54]
Fascia	7.45×10^−4^	6.40	0.40	
Skin	8.07×10^−4^	8.30	0.40	

Silicon rubbers have a wide range of hardness (10–80 HA), and are often used in cushioning and shock absorption due to their good deformation. Based on the elastic modulus scales of soft tissues, we selected silicon rubbers with hardness of 15, 35 and 55 HA to simulate the digital cushions, fascia and skin of the ostrich foot, respectively.

**Fig 2:**
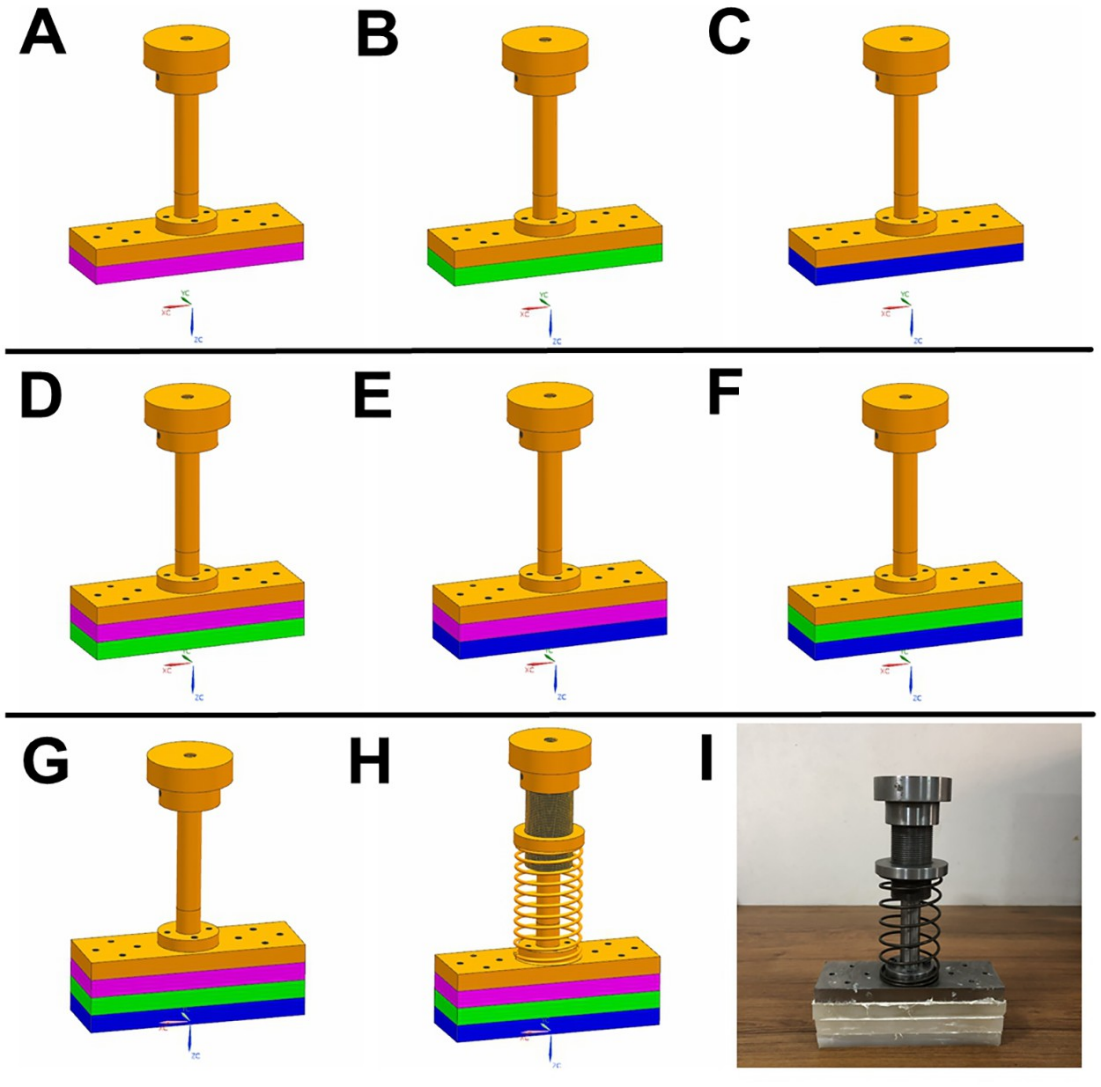
Design of different bio-inspired feet. Yellow (45# steel plate): the phalange layer; pink (15 HA silicon rubber): the digital cushion layer; green (35 HA silicon rubber): the fascia layer; blue (55 HA silicon rubber): the skin layer. (A—C), (D—F), (G): bio-inspired feet assembled with single-, double-, and three-layer silicon rubbers, respectively; (H) the bio-inspired foot added with spring (stiffness coefficient = 6 N∙mm^-1^) to simulate the functions of MTP joint; (I) a bio-inspired foot used in the tests.

Inspired by the assembly order of ostrich plantar soft tissues, we designed fourteen bio-inspired feet to study how each material layer and the assembly order of materials with different hardness degrees will affect the cushioning performance (Fig 2). The bio-inspired feet were divided into single-layer (Fig 2A–2C), double-layer (Fig 2D–2F) and three-layer (Fig 2G) assembly modes. Silicon rubber plates with different hardness degrees were glued together. In addition, a spring mechanism capable of vertically moving up and down (Fig 2H) was added to the bio-inspired foot (Fig 2G) to simulate the functions of the MTP joint.

Based on the sizes of ostrich feet, we designed the length, width and thickness of each bio-inspired foot to be 180, 60 and 60 mm, respectively [22]. The whole thickness was evenly divided into four layers. The whole mass of the final bio-inspired foot was 6.5 kg (Fig 2I).

### Setting of FEM simulation

#### FEM model establishment and material parameters

The bio-inspired foot model was imported into ABAQUS 2016, and the phalange layer was set as a rigid body. The material parameters of silicon rubbers were shown in Table 2. Simulations on loose sand were conducted with a Drucker-Prager elastoplastic model [55,56], and the material parameters were shown in Table 3.

**Table 2:** Material parameters of silicon rubbers. The densities, elasticity moduli (E) and Poisson's ratios of silicon rubbers with different hardness degrees.

Hardness (HA)	Density (g∙mm^-3^)	Elastic modulus (MPa)	Poisson's ratio
15	1.22×10^−3^	0.56	0.47
35	1.24×10^−3^	1.4	0.47
55	1.25×10^−3^	3.0	0.47

Elastic modulus (MPa) was $E=\frac{15.75+2.15H}{100-H}$, where *H* was hardness (HA).

**Table 3:** Material parameters of quartz sand. Intrinsic parameters and Drucker- Prager hardening parameters.

Intrinsic parameters		Drucker-Prager hardening parameters	
		Yield stress (MPa)	Abs plastic strain
Density (t∙m^-3^)	1.9	0.138	0
Elastic modulus (MPa)	689	0.172	0.005
Poisson's ratio	0.23	0.434	0.01
Angle of friction (°)	14.56	0.896	0.02
FlowStress ratio	1.0	1.655	0.03
Dilation angle (°)	0	2.758	0.04
-	-	4.137	0.05
-	-	6.895	0.06
-	-	8.537	0.1

#### Mesh generation and contact definition

Each layer in a bio-inspired foot on loose sand was meshed using C3D8R Hex meshes in size of 2×2×2 mm^3^. The 8-node hexahedral linear reduced integration element was chosen for fine mesh division, easy convergence in the calculation of large deformation problems, accurate displacement results and less calculation time. During the impact simulation, the phalangeal layer, the digital cushion layer, the fascia layer and the skin layer were bonded together as a whole by contact ties. Then the surface-to-surface contact between the foot and the ground, the penalty function contact in the tangential direction (friction coefficient = 0.3), and the hard contact in the normal direction were prescribed.

#### Steps and boundary conditions

Impact was simulated with the finite element method employing an explicit solver (Abaqus/Explicit, Dassault Systemes). The peak acceleration and total deformation were measured by setting the reference point at the COM of the phalange layer. In addition, the deformation of each layer, including the digital cushion layer, the fascia layer and the skin layer, was separately calculated to quantitatively compare the stabilities of different bio-inspired feet. At each time, the tested bio-inspired foot was assigned with different impact speeds in the predefined field, and the time of Step 1 was set to be 0.02 s. The whole model was subjected to the gravity of 9.8 m·s^-2^. The ground bottom was fixed and the bio-inspired foot can only vertically move up and down throughout the simulation. Deformation and stress distribution pictures were all captured at the moment of peak acceleration.

#### Sensitivity analyses

During the sensitivity analyses, the elastic modulus of the tissues was graded from the initial E_0_ (Table 1) to 0.1E_0_, E_0_ and 10E_0_, while the stiffness coefficient of the spring was graded from the initial K_0_ = 100 N·mm^-1^ to 0.1K_0_, K_0_, 10K_0_ and 100K_0_.

### Impact tests

#### Test devices

**Fig 3:**
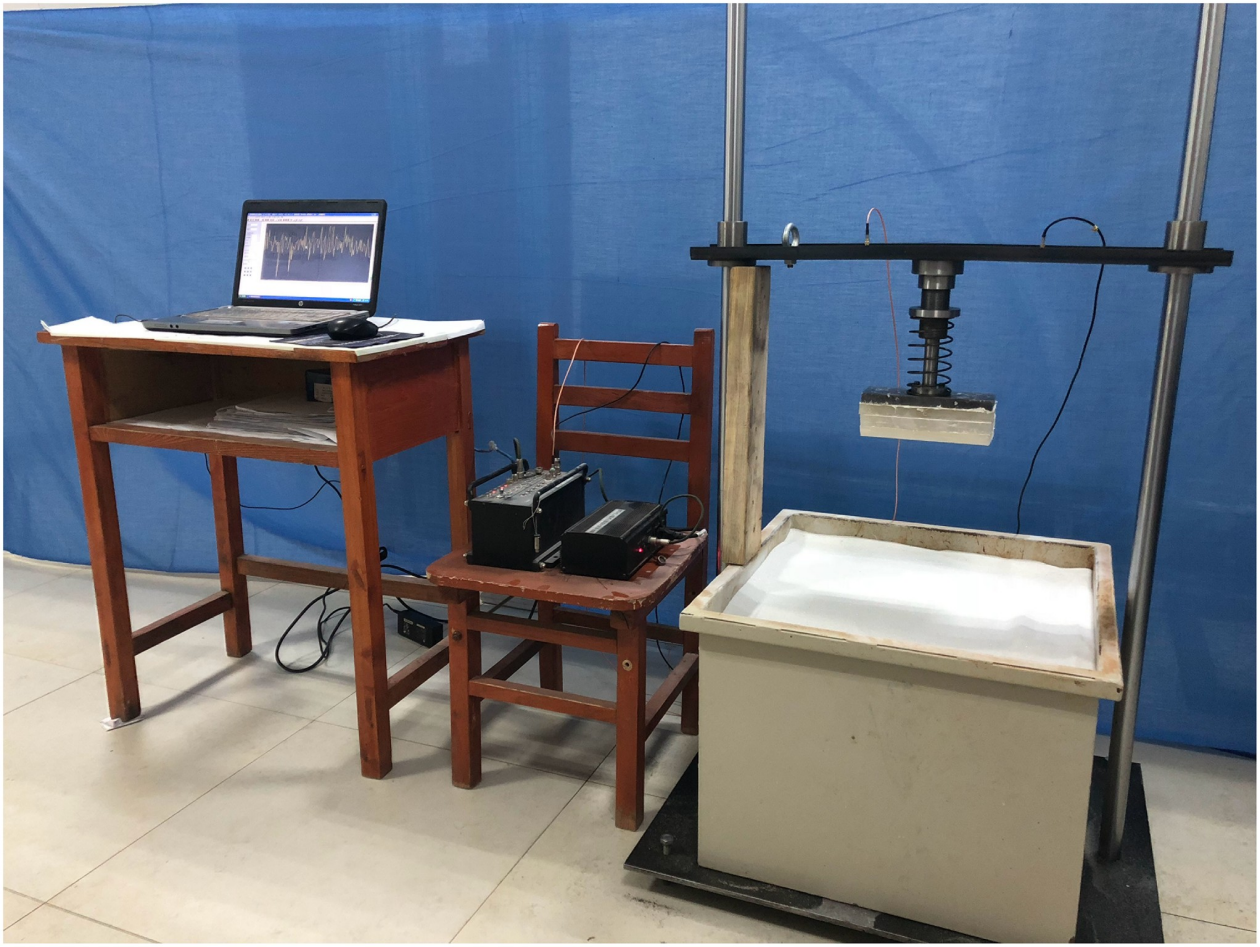
Impact test devices. Impact test bench, sand box, and a data acquisition and control system.

A 1.8-m-high impact test bench mainly consisting of two parallel polished rods, beams, and a soil box (0.5×0.5×0.5 m^3^) was self-made (Fig 3). The bench can reach an effective impact distance of 1.0 m. A linear bearing was installed in between the beam and the polished rod to reduce the friction-caused test errors. During each test, the tested bio-inspired foot bolted onto the beam can vertically move up and down along the parallel polished rods.

#### Test media

During the cushioning performance tests, white quartz sand which simulated the soft ground was uniformly distributed in the soil box to ensure smooth surface. Moreover, the solid ground was simulated using 45# steel plates.

#### Test process

The potential degradation of each layer may result in systematic error propagation. Thus, the bio-inspired feet with single-layer, double-layer, and three-layer material assemblies were successively tested after these fourteen bio-inspired feet being made. Since the maximum aboveground height of a running ostrich foot is 40 cm [32], the impact height of each bio-inspired foot was divided into 7 levels from 10 to 70 cm at the increment of 10 cm. The distance from the foot bottom to the quartz sand was measured by a ruler, and the tested bio-inspired foot freely fell along the polished rods under gravitation. Five tests were repeated at each height, and the quartz sand for each test was reprepared. We did not account for medium volume fraction in the experiments, but used a uniform filling method instead. A shovel was used to distribute the quartz sand evenly in the soil box, and then the sand surface was flattened with a scraper.

#### Data acquisition and processing

A data acquisition and control system consisting of acceleration sensors (CA-YD-180C, 0.509 mV/(m∙s^-2^)), a mobile data acquisition device (MDR-81) and a personal computer was used here. The left and right sides of the beam were each installed with two acceleration sensors, which collected and verified the acceleration data, respectively. The sampling frequency of the data acquisition device was 256 Hz, and its acquisition time was 8 s. The mean and standard deviation of peak accelerations were calculated to compare the cushioning performances among different bio-inspired feet.

Two-way analysis of variance was carried out on Origin 9.1 (OriginLab Corporation, Northampton, MA, USA) at the significance level of P < 0.05. The effects of hardness and thickness on the cushioning performance were analyzed at different impact heights on either loose sand or solid ground.

## Results

### FEM analyses

**Fig 4:**
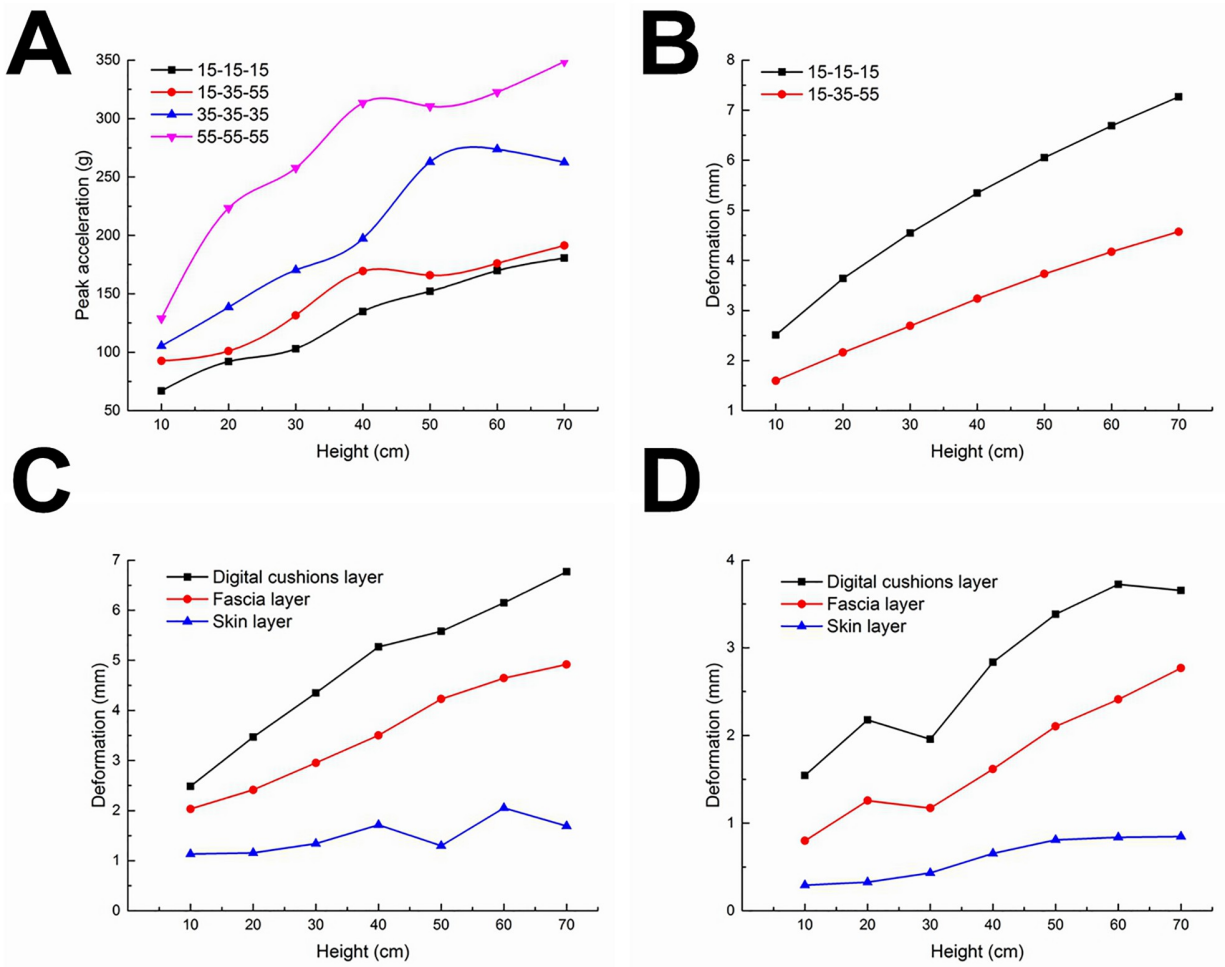
Quantitative comparisons of cushioning performances and stabilities among the bio-inspired feet on loose sand in the FEM simulations. (A) Cushioning performance, (B) total deformation; single-layer deformation of (C) 15-15-15 HA and (D) 15-35-55 HA bio-inspired feet.

The comparison of cushioning performances among the three-layer silicon rubber bio-inspired feet on loose sand is shown in Fig 4A. The peak accelerations of the 15-35-55 HA bio-inspired foot are in between those of the 15-15-15 HA and 35-35-35 HA bio-inspired feet.

The comparisons of total deformations of plantar silicon rubbers are shown in Fig 4B. The deformations of the two types of bio-inspired feet are gradually intensified with the increasing impact height. The increment of deformation gradually decreases since the deformation capacity of silicon rubbers is limited. However, the 15-35-55 HA bio-inspired foot generally deforms less than the 15-15-15 HA bio-inspired foot.

For the 15-15-15 HA (Fig 4C) and 15-35-55 HA (Fig 4D) bio-inspired feet, the deformations both rank as the digital cushion layer > the fascia layer > the skin layer, but the deformations of the 15-35-55 HA foot are all less than the corresponding layers of the 15-15-15 HA foot.

**Fig 5:**
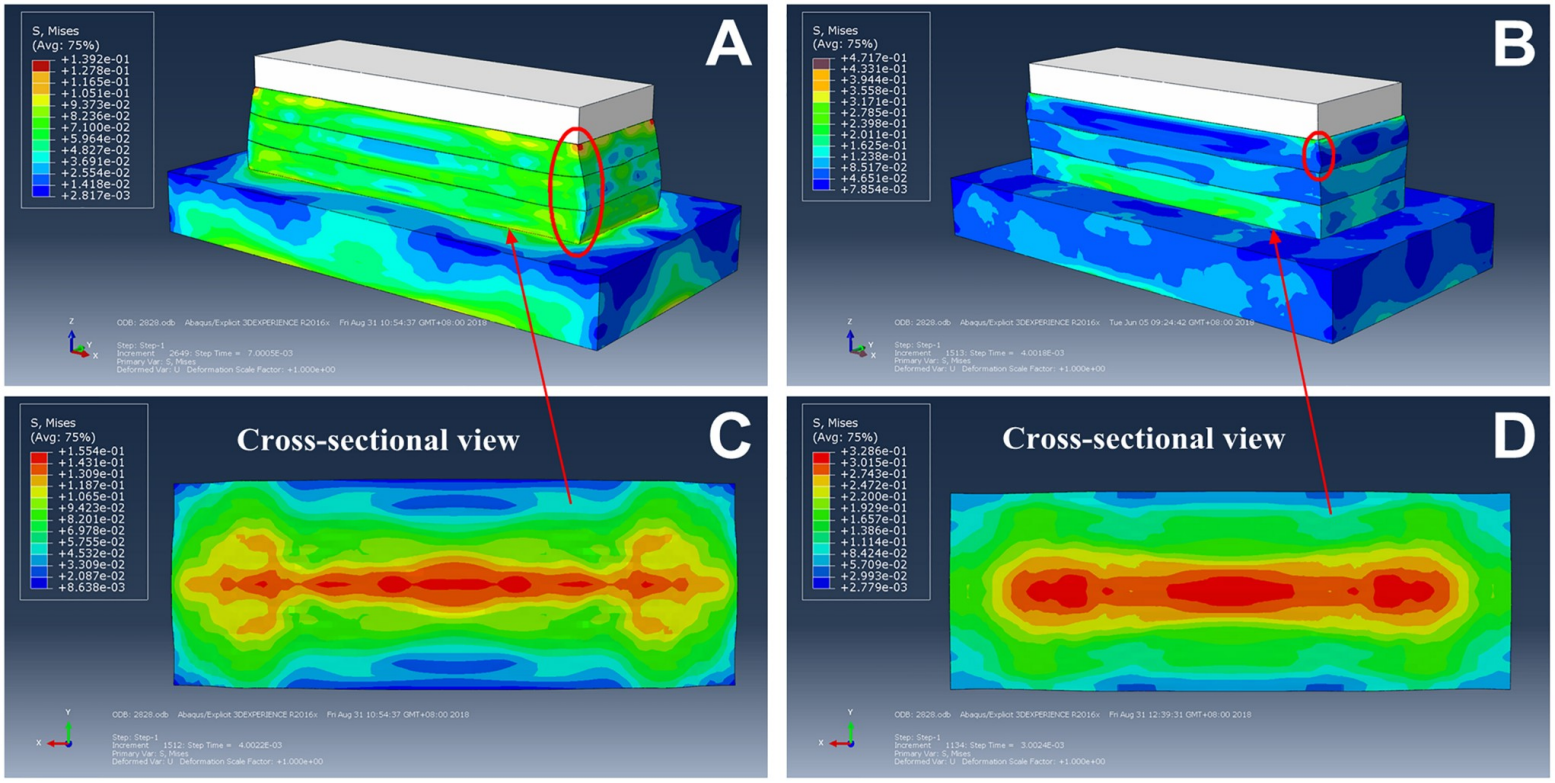
Qualitative comparisons of stress distributions and deformations between the bio-inspired feet on loose sand. Deformations and stress distributions of the 15-15-15 HA bio-inspired foot (A, C, respectively) and 15-35-55 HA bio-inspired foot (B, D, respectively).

The stress distributions of the 15-15-15 HA bio-inspired foot on loose sand at the impact height of 40 cm are shown in Fig 5A. These three layers of the bio-inspired foot shows significantly more uniform stress distributions and more obvious deformations indicated by the elliptical loop. As for the 15-35-55 HA bio-inspired foot (Fig 5B), the stress ranks as 55 HA > 35 HA > 15 HA silicon rubbers, but obvious deformation indicated by the elliptical loop only occurs in the 15 HA silicon rubber.

Fig 5C and 5D show the plantar surface stress distributions of the whole bio-inspired foot. Larger plantar surface stress (red part) is relatively irregular and concentrated at the two ends and the middle of the 15-15-15 HA bio-inspired foot, and the high stress area is thwartwise “I” type (Fig 5C), but is uniformly concentrated in the middle of the 15-35-55 HA bio-inspired foot, and the high stress area is line-like shape (Fig 5D).

Combining the results of Figs 4 and 5, the 15-35-55 HA bio-inspired foot is more stable than the 15-15-15 HA bio-inspired foot.

### Sensitivity analyses

**Fig 6:**
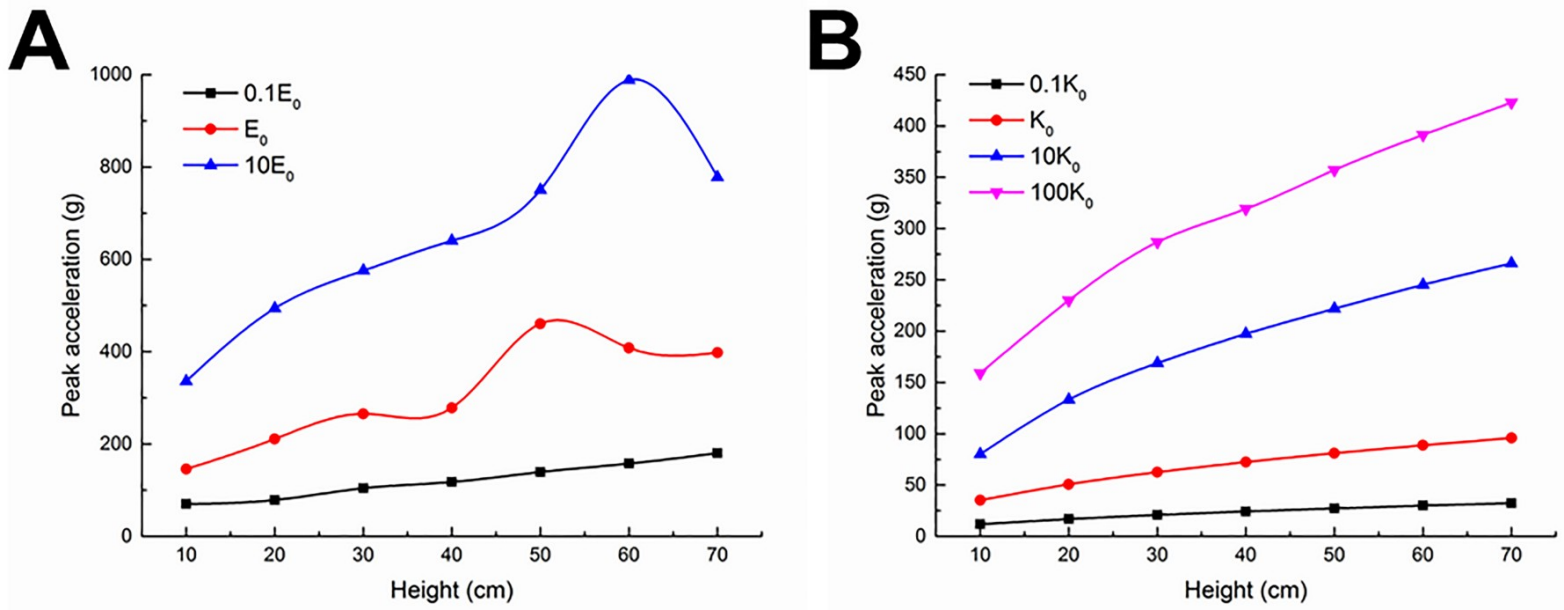
Results of sensitivity analyses in the FEM simulations. Effects of (A) elastic modulus and (B) spring stiffness coefficient.

The effects of elastic modulus (0.1E_0_, E_0_, 10E_0_) and spring stiffness coefficient (0.1K_0_, K_0_, 10K_0_, 100K_0_) on the cushioning performance of the bio-inspired feet on loose sand are shown in Fig 6A and 6B, respectively. The peak accelerations of the bio-inspired feet at different impact heights minimize at 0.1E_0_ or 0.1K_0_.

The cushioning performances of a bio-inspired foot at 0.1E_0_ and 0.1K_0_ are shown in Table 4, which show the peak accelerations of the bio-inspired foot are still larger than 10 g. However, at E_0_ and less than 10 cm of impact height, the peak accelerations at the spring stiffness coefficient of 2 and 5 N·mm^-1^ are about 5.5 and 8.5 g, respectively.

**Table 4:** Cushioning performance of the bio-inspired foot. Peak accelerations of the bio-inspired foot with specific elastic modulus and spring stiffness at different heights on loose sand.

Height (cm)	0.1K_0_ & 0.1E_0_ (g)	K = 5 N∙mm^-1^ & E_0_ (g)	K = 2 N∙mm^-1^ & E_0_ (g)
10	11.3939	8.5231	5.5972
20	16.7830	12.1960	7.8895

### Cushioning performance comparisons in the tests

**Fig 7:**
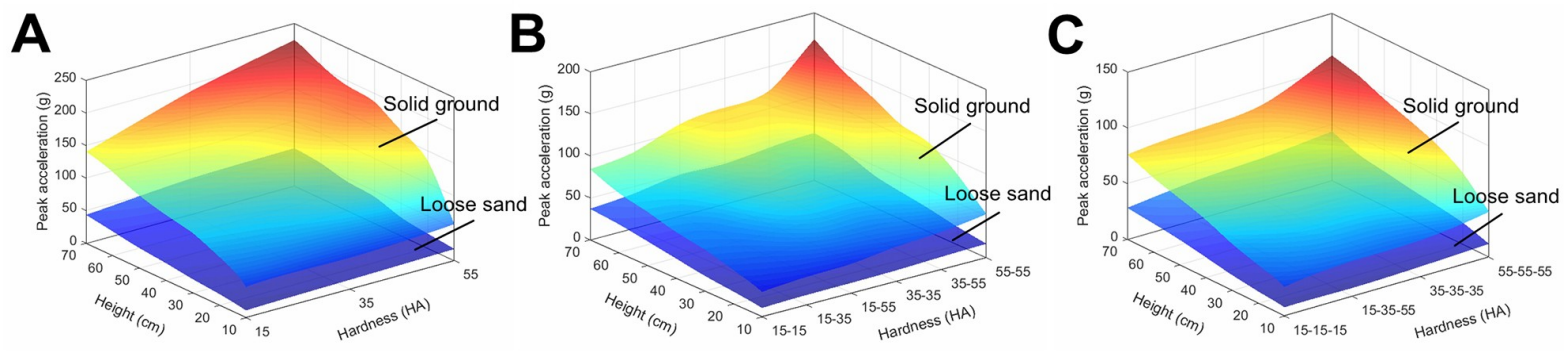
Results of impact tests. Comparisons of cushioning performances among bio-inspired feet assembled with (A) single-layer, (B) double-layer and (C) three-layer silicon rubbers on loose sand and solid ground.

The peak accelerations of the single-layer bio-inspired feet increase with the increment of impact heights, and rank as 15 HA < 35 HA < 55 HA bio-inspired feet (Fig 7A), indicating the 15 HA bio-inspired foot has the best cushioning performance. Moreover, the peak accelerations of the bio-inspired feet on loose sand are smaller than those on solid ground, which is because of the dissipatability of sand [57,58]. When the bio-inspired feet touch the loose sand, the impact or collision forces will be absorbed by quartz sands, which reduce the peak impact force.

The peak accelerations and changing rules of the double-layer bio-inspired feet are shown in Fig 7B. The peak accelerations of the 15–35 HA bio-inspired foot are smaller compared with the 15–55 HA and 35–55 HA bio-inspired feet. In addition, the peak accelerations of the 15–15 HA bio-inspired foot are smaller than the 35–35 HA and the 55–55 HA bio-inspired feet on either loose sand or solid ground, and only increase with thickness, but not with hardness.

As for the assembly of three-layer silicon rubbers, the peak accelerations of the 15-35-55 HA bio-inspired foot lie in between the 15-15-15 HA and 35-35-35 HA bio-inspired feet (Fig 7C). The changing rules of peak accelerations are not different between solid ground and loose sand.

The peak accelerations (*y*, g) of the 15-15-15 HA and 15-35-55 HA bio-inspired feet on loose sand obey Eqs (1) and (2) respectively:

$$y=0.357x+3.864\;\left({\text{R}}^{2}=0.99\right)$$ (1)

$$y=0.433x+4.220\;\left({\text{R}}^{2}=0.98\right)$$ (2)

where *x* is the impact height (cm). Obviously, Eq (2) has a larger slope on loose sand than that of Eq (1).

The peak accelerations of the 15-15-15 HA and the 15-35-55 HA bio-inspired feet on solid ground obey Eqs (3) and (4) respectively:

$$y=0.826x+17.833\;\left({\text{R}}^{2}=0.99\right)$$ (3)

$$y=0.807x+25.731\;\left({\text{R}}^{2}=0.99\right)$$ (4)

The slopes of equations on solid ground are approximately equal between the 15-15-15 HA and 15-35-55 HA bio-inspired feet.

The changing rules and cushioning performance comparisons of three-layer silicon rubbers in the FEM simulations (Fig 4A) are consistent with the experimental results (Fig 7C), except that the peak accelerations differ between the two methods. The main reason may be that the material parameters of quartz sand were directly cited from some published papers, and the loose sand in FEM was simulated using a Drucker-Prager elastoplastic model. Due to the limitation of test conditions, we did not test relevant parameters of quartz sand to avoid blind repeated tests.

In summary, the 15-15-15 HA versus the 15-35-55 HA bio-inspired foot shows better cushioning performance on both loose sand and solid ground.

### Effects of both thickness and hardness on cushioning performance

**Fig 8:**
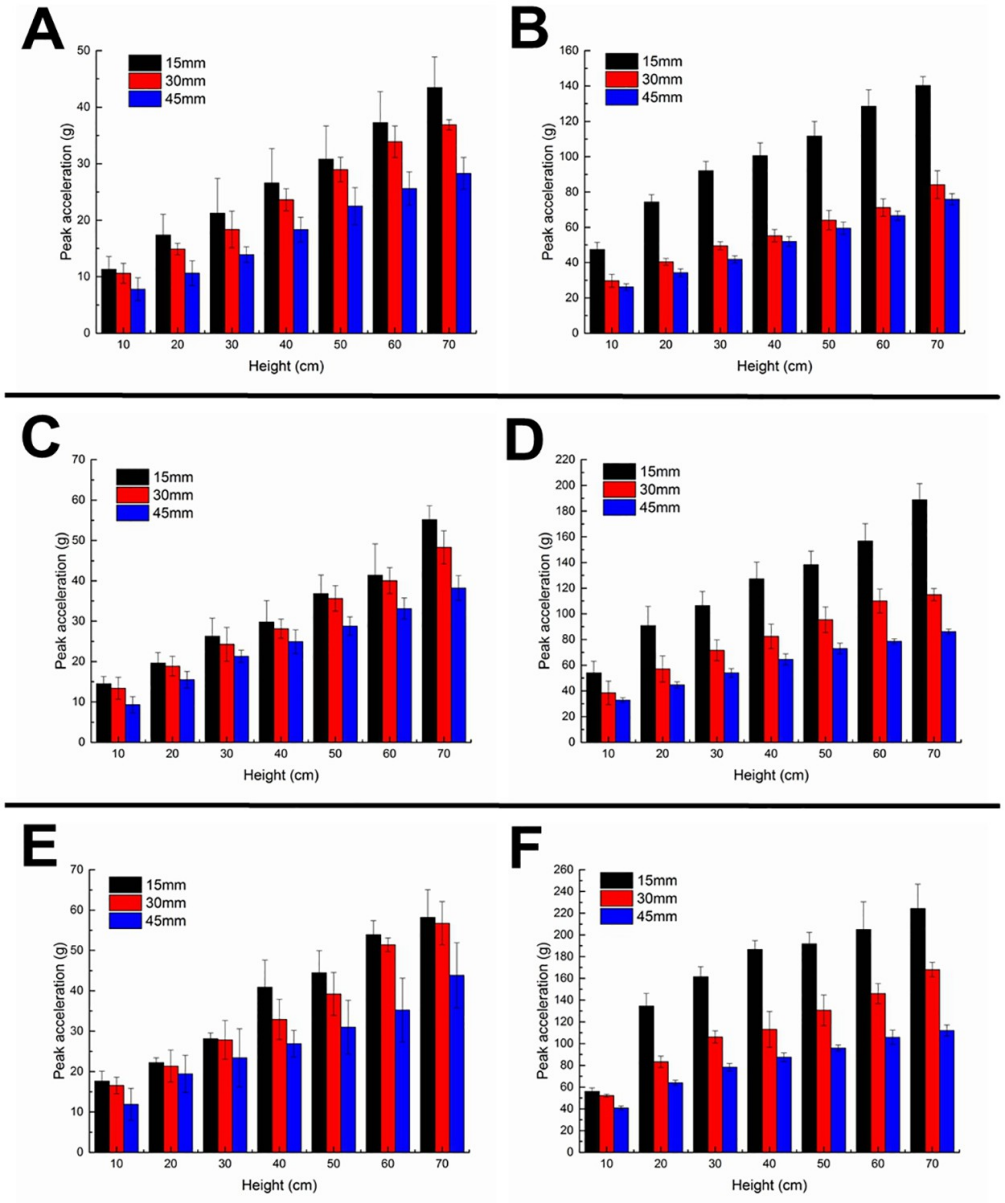
Effect of thickness on cushioning performance in the tests. Cushioning performances of 15, 35, 55 HA silicon rubbers with different thicknesses on loose sand (A, C, E, respectively) and solid ground (B, D, F, respectively).

The peak accelerations of the 15 HA bio-inspired foot on loose sand decrease with the increase of thickness at different impact heights (Fig 8A). Obviously, the peak accelerations at the thickness range from 30 to 45 mm decrease more than that at the range from 15 to 30 mm. The changing rules of peak accelerations are similar between solid ground (Fig 8B) and loose sand. However, the peak accelerations in the thickness range from 15 to 30 mm decrease more than in the range from 30 to 45 mm.

The effect of thickness on cushioning performance is similar between the 35 HA or 55 HA bio-inspired foot and the 15 HA bio-inspired foot (Fig 8C–8F). Quantitative analyses of the effect of thickness on the cushioning performance are shown in S1 Table.

Compared with those on hard ground (Fig 8B, 8D and 8F), the standard deviations of peak accelerations are relatively larger on sand ground at different heights (Fig 8A, 8C and 8E). We infer the main reason is owing to the elastoplasticity, solidifiability and dissipatability of quartz sand. Due to the fluidity, instability and porosity of quartz sand, some unexpected test errors existed in the preparation of quartz sand.

At the silicon rubber thickness of 15 mm, the peak accelerations of the bio-inspired feet on loose sand increase with the increasing hardness (S1A Fig). Similar results can be found at the silicon rubber thickness of 30 or 45 mm (S1C and S1E Fig). In addition, the results on solid ground are shown in S1B, S1D and S1F Fig, respectively, and the quantitative analyses are shown in S2 Table.

When the thickness is 45 mm and the hardness is 55 HA (Fig 8E), the fluctuations of peak accelerations of cushioning foot are enlarged with the increasing impact height. It also indicates that with the thicker and harder silicone rubber, the peak acceleration of the cushioning foot will fluctuate more severely, and the cushioning performance will be greatly affected. Therefore, we further performed significance analyses of both hardness and thickness.

### Significance analyses of both hardness and thickness

Significance analyses show both hardness and thickness are two significant influence factors on the cushioning performance, irrespective of loose sand or solid ground (Table 5). However, the effect of hardness is more significant than that of thickness on loose sand, because the P values of hardness are all smaller than those of thickness at different heights.

**Table 5:** Significant analyses of both hardness and thickness. F and P values of both hardness and thickness at different heights on loose sand and solid ground.

Height (cm)	Loose sand						Solid ground					
	Hardness		Thickness		Interaction		Hardness		Thickness		Interaction	
	F	P	F	P	F	P	F	P	F	P	F	P
10	30.42	2.66×10^−10^	26.35	2.59×10^−9^	0.66	0.62	80.36	6.55×10^−15^	93.50	5.55×10^−16^	6.73	2.91×10^−4^
20	25.31	2.31×10^−8^	12.45	3.94×10^−5^	0.94	0.45	147.14	0	190.70	0	5.94	7.28×10^−4^
30	21.51	1.45×10^−7^	7.97	9.43×10^−4^	0.26	0.90	371.38	0	424.50	0	13.35	4.21×10^−7^
40	28.27	2.24×10^−9^	21.80	7.67×10^−8^	1.86	0.13	228.05	0	296.33	0	13.43	2.25×10^−7^
50	29.17	4.02×10^−9^	27.72	7.94×10^−9^	0.74	0.57	229.42	0	304.20	0	10.98	2.27×10^−6^
60	43.06	7.83×10^−12^	42.12	1.12×10^−11^	2.14	0.09	107.51	6.66×10^−16^	165.22	0	4.56	0.0044
70	67.33	8.77×10^−15^	52.50	6.54×10^−13^	0.64	0.64	282.97	0	464.19	0	21.06	4.45×10^−10^

### Cushioning performance of the bio-inspired foot with spring

**Fig 9:**
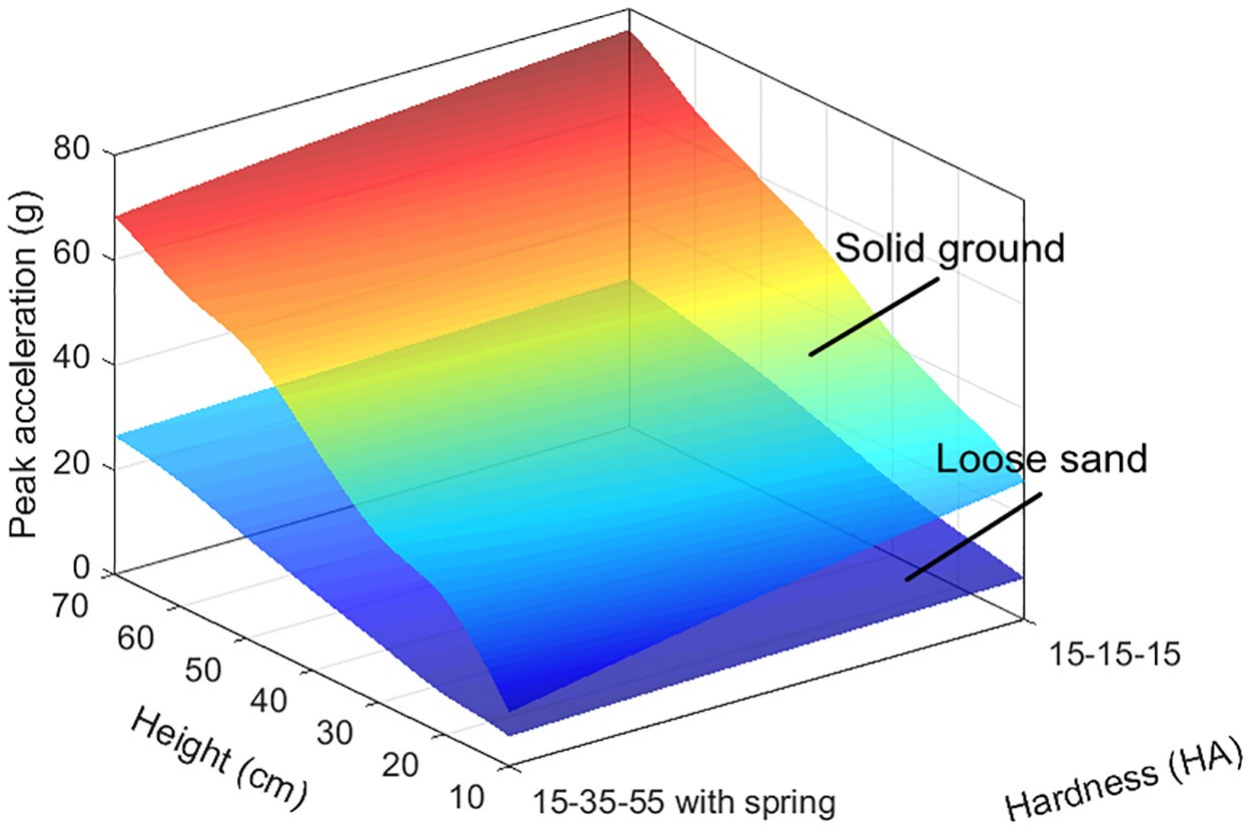
Cushioning performance of the 15-35-55HA bio-inspired foot with spring on loose sand and solid ground in the tests.

The 15-35-55 HA bio-inspired foot with spring shows less peak accelerations and thereby better cushioning performance than the 15-15-15 HA bio-inspired foot, irrespective of loose sand or solid ground (Fig 9).

## Discussion

Adult ostriches can grow up to 90–120 kg [16,26], and the weight loaded on each hindlimb even can reach 45–60 kg. The 3rd toe of an ostrich foot is only about 180 mm long and 60 mm wide [22], but has to bear the huge body weight, which requires that the ostrich foot should have excellent cushioning and shock absorption performances. In this study, design of the bio-inspired feet was inspired by the ostrich foot. The elasticity modulus scales of the skin, fascia and digital cushions were simulated from silicon rubbers with different hardness degrees and assembled into bio-inspired feet according to the assembly order of soft tissues. The impact tests showed the 15-35-55HA bio-inspired foot cushioned worse, but more stably than the 15-15-15 HA bio-inspired foot.

Ostriches are terrestrial birds with high-speed running capacity and live in deserts throughout the year. As the main actuator of high-speed running, the skin is the first to touch the ground and thus should have certain hardness and wear resistance, which is similar to the “surface hardening” treatment of mechanical parts. This treatment can improve the wear resistance and fatigue resistance, while maintaining the high toughness, strength and impact resistance in the core parts [59]. The digital cushions of the ostrich foot, which play a major role in protecting the phalanges, should bear the smallest stress. Moreover, the fascia flexibly connects and wraps the digital cushions and skin, which further compacts the internal composition of the entire ostrich foot.

During ostrich locomotion, the MTP joint that is permanently elevated aboveground plays significant roles in energy storage and shock absorption, which are similar to the spring functions [19,26,28,29]. Therefore, after studying the cushioning performances of the bio-inspired feet assembled with silicon rubbers of different hardness degrees, we designed a spring mechanism to simulate the functions of the MTP joint. The peak accelerations of the 15-35-55 HA bio-inspired foot were significantly reduced after the addition of the spring mechanism.

## Conclusions

The 15-15-15 HA bio-inspired foot showed lower peak acceleration and thereby better cushioning performance, but larger deformation, less-uniform stress distributions and thereby lower stability than the 15-35-55 HA bio-inspired foot. The silicon rubbers with different hardness degrees (which simulate the elasticity modulus scales of the digital cushions, fascia and skin), and the spring mechanism (which simulates the functions of the MTP joint), work as an “integrated system” with the cushioning and shock absorption functions.

## Perspective

In this study, we focused on how ostrich feet dealt with the ground impact and tried to study it from the engineering perspective. The bio-inspired feet were designed from the perspective of similar functions of ostrich feet. We aimed to build a bridge between biology research and engineering application and apply the excellent cushioning principle of ostriches into robot foot used in the soft ground. Therefore, some bio-inspired feet were designed from that case.

Our current bio-inspired feet are not a close match to ostrich feet. As we all know, ostrich feet are fascinating structures with complex assemblies. We are unable to find alternatives to ostrich foot muscles, tendons, joints and ligaments in reality, and cannot replicate biological feet. In addition, we currently pay more attention to the cushioning and shock absorption performances of different mechanical feet in the vertical direction, without considering the three-dimensional dynamics of a running animal. For example, a biped will decelerate after foot touchdown and accelerate again before liftoff in each step during locomotion, creating not only a vertical force, but also a strong force against the travel direction at touchdown. The muscle-tendon force will much surpass the ground reaction force due to the small moment arms of the tendons crossing the MTP joint. The internal forces are very complicated, and we cannot achieve this goal in the view of engineering applications at present. Therefore, we tried to simplify the ostrich foot from its structures, joint functions, assembly order and elasticity modulus scales of the soft tissues and thereby to study whether our research methods were effective or what effects they would have.

This study can be considered as a preliminary one. If the suitable substitutes for biomaterial, coupling connection mode, functional mechanism and an impact device for simulating the touchdown movement of a running animal will be found, we will further study them.

## Supporting information

S1 Fig Effect of hardness on cushioning performance in the tests. Cushioning performances of 15-, 30-, 45-mm-thick silicon rubbers with different hardness degrees on loose sand (A, C, E, respectively) and solid ground (B, D, F, respectively).
(TIFF)

S1 Table Quantitative analyses of the effect of thickness on cushioning performance. Decrements of peak accelerations of 15, 35, 55 HA silicon rubbers with different thicknesses on loose sand and solid ground.
(DOCX)

S2 Table Quantitative analyses of the effect of hardness on cushioning performance. Increments of peak accelerations of 15-, 30-, 45-mm-thick silicon rubbers with different hardness degrees on loose sand and solid ground.
(DOCX)

S1 Dataset Raw data for tests and FEM simulations. Figs 4, 6, 7 and 9 were drawn using these raw data.
(XLSX)
