# Supplementary figures and images for: Study on bio-inspired feet based on the cushioning and shock absorption characteristics of the ostrich foot
Source: PLoS One. 2020 Jul 24;15(7):e0236324. doi: 10.1371/journal.pone.0236324 (PMC7380592; doi:10.1371/journal.pone.0236324)

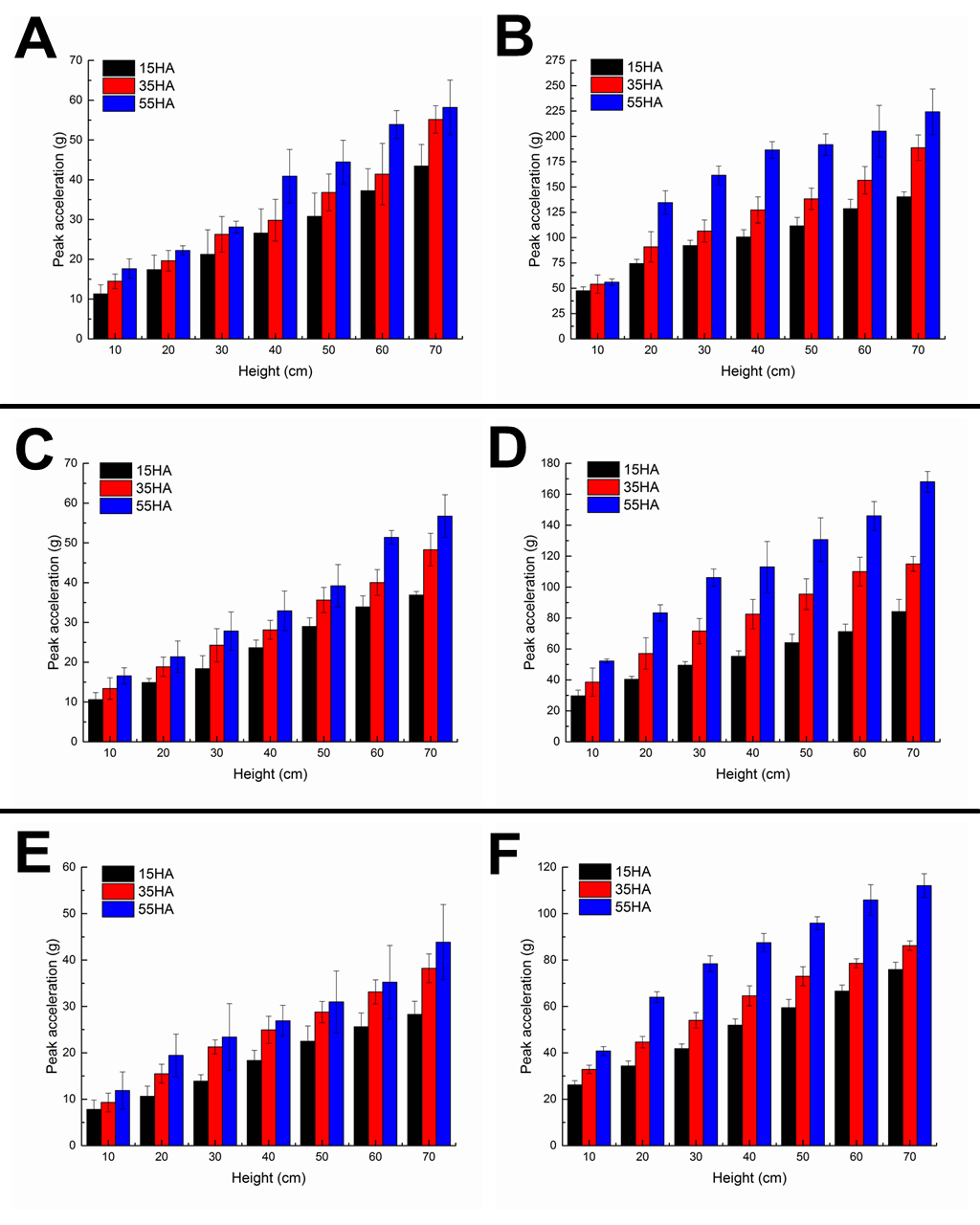

Supplement: S1 Fig — Cushioning performances of 15-, 30-, 45-mm-thick silicon rubbers with different hardness degrees on loose sand (A, C, E, respectively) and solid ground (B, D, F, respectively). (TIFF) [file pone.0236324.s001.tiff]
