# Supplementary material for: Study on bio-inspired feet based on the cushioning and shock absorption characteristics of the ostrich foot
Source: PLoS One. 2020 Jul 24;15(7):e0236324. doi: 10.1371/journal.pone.0236324 (PMC7380592; doi:10.1371/journal.pone.0236324)
Supplement: S1 Table — Decrements of peak accelerations of 15, 35, 55 HA silicon rubbers with different thicknesses on loose sand and solid ground. (DOCX) [file pone.0236324.s002.docx]

| Hardness (HA) | Medium | Thickness (mm) | Height (cm) | | | | | | | Means ± s.d. |
| --- | --- | --- | --- | --- | --- | --- | --- | --- | --- | --- |
|  |  |  | 10 | 20 | 30 | 40 | 50 | 60 | 70 |  |
| 15 | Loose sand | 15 to 30 | 6.0 | 14.4 | 13.5 | 11.1 | 5.9 | 9.0 | 15.1 | 10.7 ± 3.8 |
|  |  | 30 to 45 | 26.5 | 28.7 | 24.3 | 22.4 | 22.3 | 24.4 | 23.3 | 24.6 ± 2.3 |
|  | Solid ground | 15 to 30 | 37.4 | 45.6 | 46.2 | 45.1 | 42.6 | 44.6 | 40.0 | 43.1 ± 3.3 |
|  |  | 30 to 45 | 11.9 | 15.1 | 15.5 | 6.0 | 7.1 | 6.5 | 9.8 | 10.3 ± 4.0 |
| 35 | Loose sand | 15 to 30 | 7.5 | 4.0 | 7.6 | 5.7 | 3.2 | 3.3 | 12.4 | 6.3 ± 3.3 |
|  |  | 30 to 45 | 30.5 | 17.8 | 12.3 | 11.4 | 19.2 | 17.3 | 20.9 | 18.5 ± 6.3 |
|  | Solid ground | 15 to 30 | 28.7 | 37.1 | 32.8 | 35.2 | 31.0 | 29.8 | 39.1 | 33.4 ± 3.9 |
|  |  | 30 to 45 | 14.7 | 21.9 | 24.5 | 21.7 | 23.5 | 28.6 | 25.0 | 22.8 ± 4.3 |
| 55 | Loose sand | 15 to 30 | 6.1 | 3.8 | 1.0 | 19.5 | 11.8 | 4.6 | 2.5 | 7.0 ± 6.5 |
|  |  | 30 to 45 | 28.2 | 9.0 | 15.9 | 18.3 | 21.0 | 31.5 | 22.7 | 20.9 ± 7.5 |
|  | Solid ground | 15 to 30 | 6.8 | 38.0 | 34.3 | 39.4 | 31.9 | 28.8 | 25.0 | 29.2 ± 11.1 |
|  |  | 30 to 45 | 21.8 | 23.2 | 26.1 | 22.6 | 26.6 | 27.5 | 33.3 | 25.9 ± 3.9 |

Notes.

The unit was %.
