# Supplementary material for: Study on bio-inspired feet based on the cushioning and shock absorption characteristics of the ostrich foot
Source: PLoS One. 2020 Jul 24;15(7):e0236324. doi: 10.1371/journal.pone.0236324 (PMC7380592; doi:10.1371/journal.pone.0236324)
Supplement: S2 Table — Increments of peak accelerations of 15-, 30-, 45-mm-thick silicon rubbers with different hardness degrees on loose sand and solid ground. (DOCX) [file pone.0236324.s003.docx]

| Thickness (mm) | Medium | Hardness (HA) | Height (cm) | | | | | | | Means ± s.d. |
| --- | --- | --- | --- | --- | --- | --- | --- | --- | --- | --- |
|  |  |  | 10 | 20 | 30 | 40 | 50 | 60 | 70 |  |
| 15 | Loose sand | 15 to 35 | 28.2 | 12.9 | 23.6 | 12.2 | 19.5 | 11.2 | 26.9 | 19.2 ± 7.2 |
|  |  | 35 to 55 | 21.9 | 13.2 | 7.0 | 37.1 | 20.7 | 30.1 | 5.5 | 19.4 ± 11.7 |
|  | Solid ground | 15 to 35 | 13.9 | 22.3 | 15.7 | 26.6 | 23.9 | 21.9 | 34.6 | 22.7 ± 6.9 |
|  |  | 35 to 55 | 3.6 | 48.1 | 51.7 | 46.5 | 38.7 | 30.9 | 18.8 | 34.0 ± 17.6 |
| 30 | Loose sand | 15 to 35 | 26.2 | 26.6 | 32.0 | 19.0 | 23.0 | 18.2 | 30.9 | 25.1 ± 5.4 |
|  |  | 35 to 55 | 23.8 | 13.4 | 14.7 | 17.0 | 10.0 | 28.4 | 17.5 | 17.8 ± 6.3 |
|  | Solid ground | 15 to 35 | 29.7 | 41.4 | 44.6 | 49.3 | 49.0 | 54.6 | 36.7 | 43.6 ± 8.5 |
|  |  | 35 to 55 | 35.5 | 46.0 | 48.3 | 37.1 | 37.0 | 32.7 | 46.2 | 40.4 ± 6.2 |
| 45 | Loose sand | 15 to 35 | 19.3 | 45.9 | 53.0 | 36.0 | 27.9 | 29.4 | 35.1 | 35.2 ± 11.4 |
|  |  | 35 to 55 | 27.8 | 25.5 | 10.0 | 7.9 | 7.6 | 6.4 | 14.8 | 14.3 ± 8.9 |
|  | Solid ground | 15 to 35 | 25.6 | 30.0 | 29.2 | 24.3 | 22.7 | 18.0 | 13.6 | 23.4 ± 5.9 |
|  |  | 35 to 55 | 24.1 | 43.4 | 45.1 | 35.5 | 31.4 | 34.7 | 30.0 | 34.9 ± 7.4 |

Notes.

The unit was %.
